# SENP1 is a crucial promotor for hepatocellular carcinoma through deSUMOylation of UBE2T

**DOI:** 10.18632/aging.102700

**Published:** 2020-01-22

**Authors:** Yifeng Tao, Ruidong Li, Conghuan Shen, Jianhua Li, Quanbao Zhang, Zhenyu Ma, Feifei Wang, Zhengxin Wang

**Affiliations:** 1 Department of General Surgery and Liver Transplant Center, Huashan Hospital, Fudan University, Shanghai 200040, China; 2 Institute of Organ Transplantation, Fudan University, Shanghai 200040, China; 3 Bioscience Research Center, Shanghai 200120, China

**Keywords:** hepatocellular carcinoma, SENP1, deSUMOylation, UBE2T, Akt

## Abstract

The cooperative roles of SENP1 and UBE2T in development and progression of hepatocellular carcinoma (HCC) are still unknown. The expression levels of SENP1 and UBE2T were evaluated in clinical specimens and HCC cells. The relationship between clinicopathological features and SENP1 were analyzed. We constructed the HepG2-SENP1 knockout cell model and explored the functions of SENP1 and UBE2T in HCC development. UBE2T was confirmed as a novel deSUMOylation target of SENP1. Upregulation of SENP1 and UBE2T were observed in HCC tissues and most hepatoma cell lines, and their expression levels were proved to be positively related. Knockout of SENP1 resulted in impaired growth, migration and invasion, and enhanced apoptosis in vitro, as well as inhibition of tumor growth in vivo. Furthermore, we demonstrated that SENP1 could directly deSUMOylate UBE2T thereby increasing its expression and activating Akt pathway. Functional studies showed that UBE2T overexpression or K8R mutation promoted cell growth, migration and invasion. In conclusion, our study demonstrated that SENP1 and UBE2T were positively related and functioned as tumor promoters. The carcinogenesis of SENP1 is mediated by deSUMOylation of UBE2T and the UBE2T/Akt pathway. Notably, UBE2T was identified as a novel deSUMOylation target of SENP1 in this study for the first time.

## INTRODUCTION

Liver cancer, especially the primary liver cancer as well as hepatocellular carcinoma (HCC), is the fifth most newly diagnosed cancer and the third leading cause for cancer-related death in the world [1]. Therefore, the development of novel or more efficient therapy or anti-cancer drugs is of great significance for HCC patients. However, in spite of the various strategies that have been developed for treating HCC such as liver transplantation, hepatectomy, radiofrequency ablation (RFA) and chemotherapy, the average 5-year survival rate for HCC patients still remains very poor and is in urgent need of improvement [2]. Although plenty of research has been focused on the complicated molecular mechanism of HCC initiation and progression, the understanding is still not fully clear. As far as we know, the development of HCC is a multi-step and gradual process, along with the disorder of the expression regulation of many tumor suppressor genes and oncogenes, such as the inactivation or decreased expression of PTEN and P53 as tumor suppressor genes, and the activation or increased expression of GPC3 and TGF-β1 as oncogenes. Nevertheless, these genes still cannot be used as molecular markers for early diagnosis and prognosis of HCC [3–6]. Therefore, it is imperative to uncover the molecular mechanism of HCC and identify new possible targets for clinical management of HCC.

SUMOylation, a reversible post-translational protein modification which could create the on-and-off state for biological regulation, involved conjugation of small ubiquitin-like modifiers (SUMOs) to target proteins [7]. SUMOylation is a dynamic process that can be catalyzed by SUMO-specific activating (E1), conjugating (E2) and ligating (E3) enzymes. In contrast to other more complex ubiquitin pathway, SUMOylation needs only a single conjugating enzyme and a limited number of ligases, which also manifests in the off step [8]. The reversal of SUMOylation is achieved by a family of SUMO-specific proteases (SENPs) [8]. Among the seven SENP members, SENP1 is widely localized in the nuclei and has been reported to play fairly important role in lung cancer [9], prostate cancer [10], colon cancer [11]. However, the role of SENP1 in HCC is still largely unknown. Moreover, accumulating evidence demonstrated that SENP1 could deconjugate a number of SUMOylated proteins, including HDAC1 [12], androgen receptor (AR) [13], HIF1α [14], Sirt1 [15], Gata1 [16], Pin1 [17] and Akt [18]. However, up to now, only a handful of SENP1 substrates have been found and identified, and its targets and molecular mechanisms during tumorigenesis are still not well understood.

Ubiquitin-conjugating enzyme E2T (UBE2T), also known as HSPC150, belongs to the E2 family and has been predicted to be an ubiquitin-conjugating enzyme because of having characteristic UBC-homology domain [19]. Increasing evidence illustrated that, as one of the important members in the ubiquitin-protease system, UBE2T is closely related to cell proliferation, apoptosis, immune response, and tumor occurrence. At present, UBE2T has been reported to be upregulated in lung cancer [20], breast cancer [21], prostate cancer [22], nasopharyngeal carcinoma [23] and HCC [24]. Moreover, further studies on its expression in breast cancer suggested that UBE2T could regulate cell proliferation and promote occurrence of tumor, suggesting its great potential as a new target for tumor therapy [21]. Combining the aforementioned information and the important role of UBE2T in genomic integrity and carcinogenesis, this study aimed to explore the relationship between SENP1 and UBE2T and their roles in development and progression of HCC.

Herein, we demonstrated that the expression levels of SENP1 and UBE2T were upregulated in human HCC tissues and UBE2T levels were positively correlated with SENP1 levels. Clinical data collected from HCC patients demonstrated that patients with higher SENP1 expression level suffered from lower survival rate. We further identified ubiquitin-conjugating enzyme E2T (UBE2T) as a novel target of SENP1 with KRE site on UBE2T as a major SUMOylation site. Furthermore, knockout of SENP1 inhibited HCC development while overexpression of UBE2T or disruption of SUMOylation of UBE2T showed the opposite effects. Thus, SENP1 may promote development and progression of HCC through deSUMOylating UBE2T. The new discovered association between SENP1/UBE2T and HCC may present a novel and effective therapeutic method for HCC.

## RESULTS

### Upregulation of SENP1 in HCC tissues

**Figure 1:**
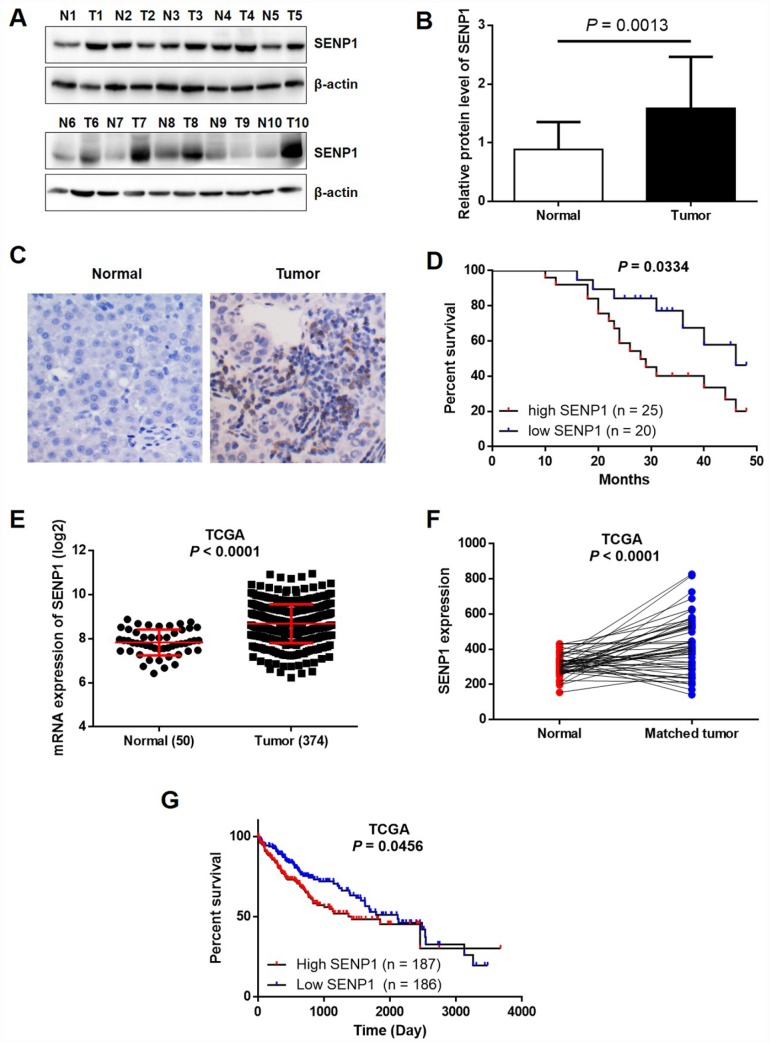
**SENP1 is overexpressed in HCC tissues.** (**A**) The protein levels of SENP1 in adjacent normal tissues and tumor tissues were examined by Western blot. (**B**) The average relative protein levels of SENP1 in adjacent normal tissues and tumor tissues obtained from gray analysis of Western blot results. (**C**) The expression of SENP1 in adjacent normal tissues and tumor tissues was determined by immunohistochemical staining. (**D**) Kaplan-Meier’s analysis of the correlation between SENP1 levels and overall survival in HCC patients. (**E**, **F**) SENP1 expression in TCGA RNAseq database. (**G**) The association between SENP1 expression and survival rate in TCGA database. The representative images were selected from at least three independent experiments.

Previously, it has been clearly reported that SENP1 is overexpressed in several types of human cancer, including colon cancer [11], prostate cancer [10], and breast cancer [25]. In order to investigate the relationship between SENP1 and HCC, the expression levels of SENP1 in HCC tissues and para-carcinoma tissues were detected by Western blot analysis and immunohistochemistry (IHC). As shown in Figure 1A and 1B, the expression level of SENP1 was obviously higher in tumor tissues than para-carcinoma tissues in the randomly selected clinical specimens. The immunohistochemical staining of both tumor and para-carcinoma tissues with SENP1 antibody showed similar results, indicating the upregulated expression of SENP1 in tumor tissues and suggesting the involvement of SENP1 in HCC progression (Figure 1C). Moreover, Kaplan-Meier analysis showed that HCC patients with relatively high SENP1 expression suffered from lower overall survival rate (Figure 1D). Notably, data mining of RNA-seq and clinical data that are publically available in The Cancer Genome Atlas (TCGA) database (https://cancergenome.nih.gov/, dataset: TCGA-LIHC) confirmed that SENP1 expression is upregulated in tumor tissues (50 normal tissues *vs.* 374 tumor tissues, or 50 normal tissues *vs.* 50 matched tumor tissues, *P* < 0.001) and associated with lower survival rate (Figure 1E–1G). Furthermore, statistical analysis was performed to reveal the association between SENP1 expression and tumor characteristics collected from 50 HCC patients. As shown in Table 1, patients with higher expression level of SENP1 generally suffered from larger and more tumors, poorer histological characteristics and later TNM stage, which was also in accordance with the results of overall survival rate.

**Table 1:** Relationship between SENP1 and tumor characteristics in 50 patients with HCC.

	**Number of cases**	**SENP1 expression**		**P-value**
		**Low**	**High**	
Sex				
Male	32	15	17	0.736
Female	18	8	10	
Age at Diagnosis (yr)				
≤50	22	13	9	0.825
>50	28	12	16	
Tumor Size(cm)				
≤5	20	14	6	0.003*
>5	30	10	20	
Histological Type				
High/moderate	25	15	10	0.113
Poor	25	9	16	
TNM stage				
I	16	5	11	0.226
II~III	34	12	22	
HBV				
Negative	20	11	9	0.732
Positive	30	14	16	
Tumor number				
Single	27	18	9	0.013*
Two or more	23	7	15	
Metastasis				
Positive	23	5	18	<0.001*
Negative	27	19	8	

Statistically significant (p<0.05). Data were analyzed by chi-squared test using SPSS 17.0. TNM: tumor-node-metastasis staging system; HBV: hepatitis B virus.

### Effects of SENP1 knockout on HCC cell growth, cell cycle distribution, cell migration and invasion

**Figure 2:**
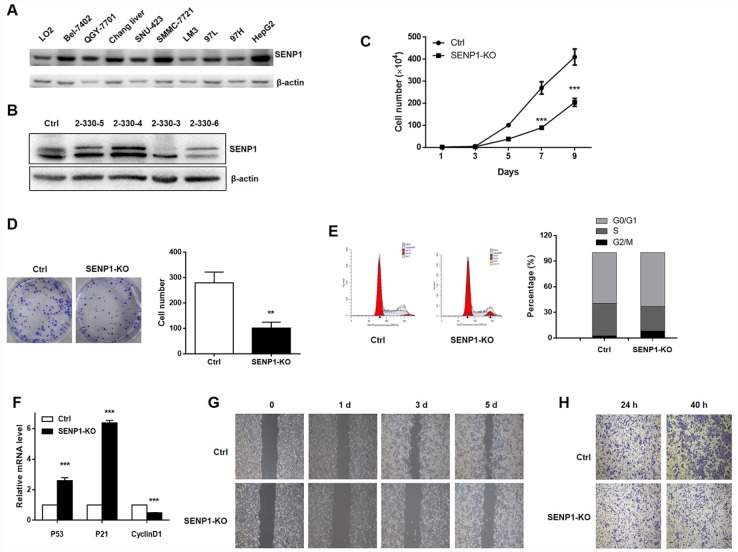
**SENP1 knockout inhibits cell proliferation and motion, and induces cell cycle dysregulation.** (**A**) The protein levels of SENP1 in normal hepatic cells and nine HCC cell lines were measured using Western blot. (**B**) SENP1 knockout was confirmed by Western blot. (**C**) The effect of SENP1 knockout on HepG2 cell proliferation was determined using cell count assay. (**D**) The effects of SENP1 knockout on HepG2 cell colony formation were examined using colony formation assay. (**E**) The effects of SENP1 knockout on HepG2 cell cycle distribution were analyzed by using flow cytometry. (**F**) The effects of SENP1 knockout on P53, P21 and CyclinD1 mRNA levels were measured using RT-PCR. (**G**) The effects of SENP1 knockout on HepG2 cell migration were determined using wound healing assay. (**H**) The effects of SENP1 knockout on HepG2 cell invasion were examined using Transwell assay. The representative images were selected from at least three independent experiments. *P<0.05, **P<0.01, ***P<0.001.

The protein level of SENP1 was examined in hepatocyte line (LO2) and hepatoma cell lines (Bel-7402, QGY-7701, Chang liver, SNU-423, SMMC-7721, LM3, 97L, 97H, HepG2). As shown in Figure 2A, most hepatoma cell lines showed fairly high expression level of SENP1, including Chang liver, SNU-423, SMMC-7721, LM3, 97H and HepG2. Considering the highest SENP1 expression in HepG2 cell line, it was selected as the cell model for the subsequent research. SENP1 knockout cells were established based on the CRISPR/Cas9 system and the successful knockout was confirmed by Western blot analysis (Figure 2B). Given that the 2-330-3 group showed the best knockout efficiency, the 2-330-3 group was utilized to perform the subsequent experiments. Cell proliferation and colony formation, which were evaluated by cell count assay and colony formation assay (Figure 2C and 2D), respectively, displayed that knockout of SENP1 significantly inhibited proliferative capacity and colony formation ability of HepG2 cells compared with the negative control. Then we detected the cell cycle distribution and demonstrated that knockout of SENP1 arrested G0/G1 phase and decreased S phase in HepG2 cells compared with negative control (Figure 2E). Moreover, as shown in Figure 2F, SENP1 knockout significantly enhanced the mRNA levels of P53 and P21, and reduced the CyclinD1 mRNA level in HepG2 cells. To further investigate the effect of SENP1 knockout on cell migration and invasion capacities, wound healing assay and Transwell assay were performed and demonstrated that SENP1 knockout could repress migration and invasion of HepG2 cells (Figure 2G and 2H).

### Upregulation of UBE2T in HCC tissues and cell lines

**Figure 3:**
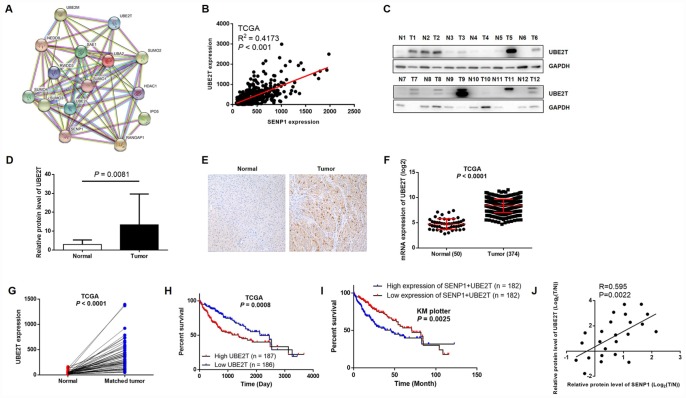
**UBE2T is overexpressed in HCC tissues and cell lines.** (**A**) SENP1-related interaction network was constructed based on String database. (**B**) Correlation analysis of SENP1 and UBE2T expression was performed based on gene expression profiling data of TCGA. (**C**) The protein levels of UBE2T in adjacent normal tissues and tumor tissues were examined by western blot. (**D**) The average relative protein levels of UBE2T in adjacent normal tissues and tumor tissues obtained from gray analysis of Western blot results. (**E**) The expression of UBE2T in adjacent normal tissues and tumor tissues was determined by immunohistochemical staining. (**F**, **G**) UBE2T expression in TCGA RNAseq database. (**H**, **I**) The association between UBE2T expression or SENP1+UBE2T expression and survival rate in TCGA database or KM plotter dataset. (**J**) Expression of SENP1 and UBE2T were positively related in HCC clinical samples. The data were analyzed with Pearson correlation by GraphPad prism 6.0. The representative images were selected from at least three independent experiments.

In order to further explore the underlying mechanism of the promotion effects on HCC by SENP1, String database was utilized to construct SENP1-related interaction network, indicating its potential association with UBE2T (Figure 3A). Furthermore, the positive correlation between expression of SENP1 and UBE2T in HCC, which was revealed based on data of TCGA, also proved the assumption (Figure 3B). Subsequently, to confirm the upregulation of UBE2T level in HCC and explore whether an association exists between SENP1 and UBE2T, Western blot analysis and immunohistochemical staining were employed to detect the expression level of UBE2T. As shown in Figure 3C, the results revealed significantly higher expression level of UBE2T in tumor tissues than para-carcinoma tissues (Figure 3D). As expected, the immunohistochemical staining showed the same trend as shown in Figure 3E. Actually, the data collected from TCGA database also proved the upregulation of UBE2T in tumor tissues (50 normal tissues *vs.* 374 tumor tissues, or 50 normal tissues *vs.* 50 matched tumor tissues, *P* < 0.001, Figure 3F and 3G). Moreover, the association between high expression of UBE2T or simultaneously high expression of UBE2T and SENP1 with lower survival rate was showed by TCGA database and KM plotter liver cancer dataset (Figure 3H and 3I). More importantly, through Pearson correlation of the corresponding expression level of SENP1 and UBE2T in tumor tissues and related para-carcinoma tissues, we also established the positive correlation between the expression level of SENP1 and UBE2T (R = 0.595, *P* = 0.0022, Figure 3J), which is also in consistent with our previous speculation.

### Effects of SENP1 knockout on its downstream

**Figure 4:**
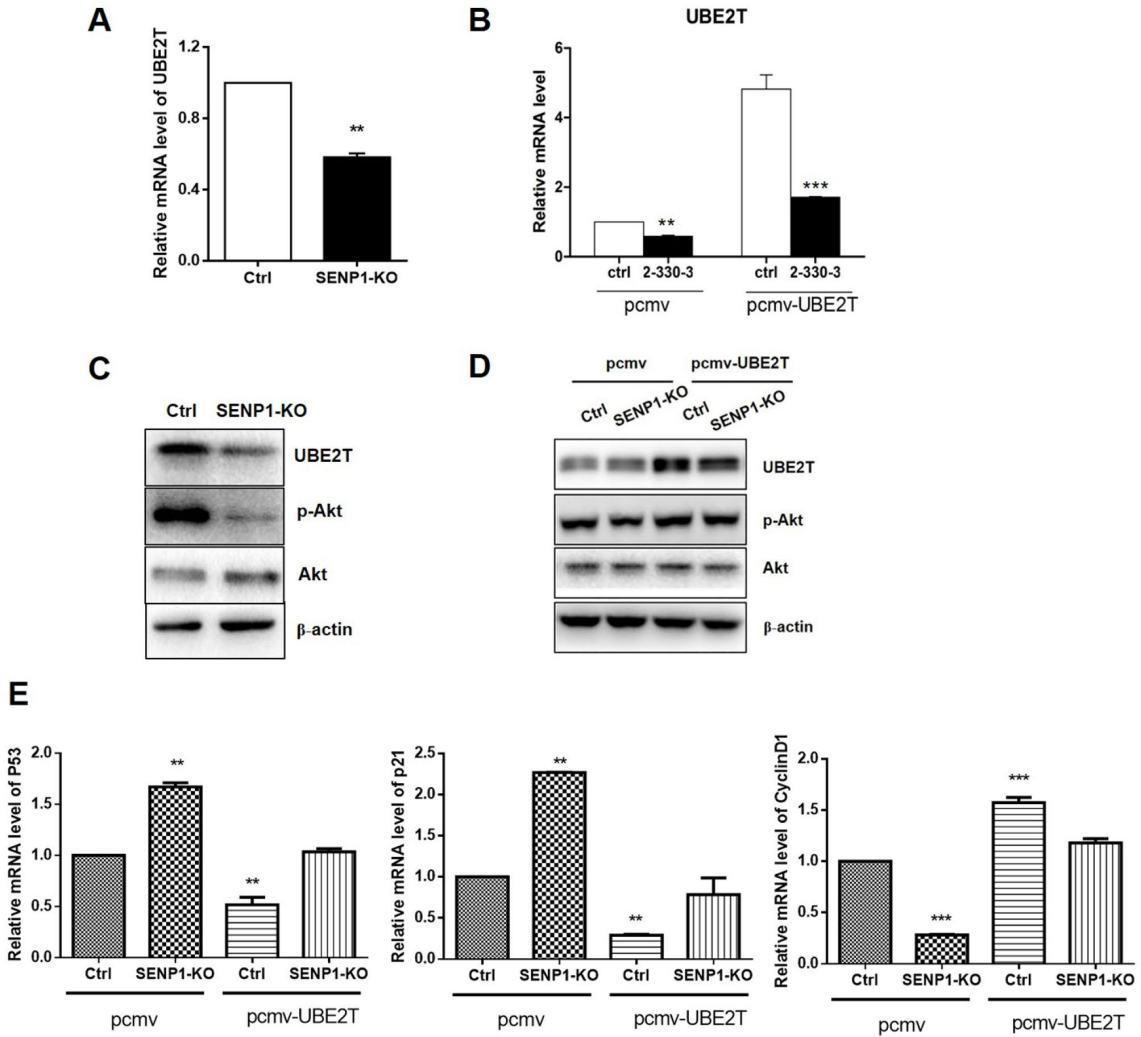
**SENP1 promotes UBE2T signaling pathway.** (**A**, **B**) SENP1 knockout inhibited the mRNA level of UBE2T, which was reversed by UBE2T overexpression. (**C**, **D**) SENP1 knockout inhibited the activation of Akt, which was reversed by UBE2T overexpression. (**E**) UBE2T overexpression weakened the effect of SENP1 knockout in P53, P21 and CyclinD1 levels. The representative images were selected from at least three independent experiments. *P<0.05, **P<0.01, ***P<0.001.

In view of the fact of UBE2T overexpression in HCC and positive correlation between SENP1 and UBE2T, we were wondering whether a direct interaction exists between SENP1 and UBE2T. Bearing these in mind, PCMV-UBE2T plasmid was constructed and co-expressed with negative control or SENP1 knockout for overexpression of UBE2T, while the vector PCMV was used as the negative control. Figure 4A–4C showed that SENP1 knockout inhibited the protein level of UBE2T, which could be reversed by UBE2T overexpression. It has been reported that UBE2T could activate Akt signaling pathway [23]. Herein, as shown in Figure 4C and 4D, activation of Akt, as well as the expression of p- Akt, was also found to be significantly inhibited by SENP1 knockout which also could be reversed by overexpression of UBE2T. As described above, SENP1 knockout significantly enhanced P53 and P21 levels, and reduced CyclinD1 levels. Herein, the opposite effect of UBE2T overexpression was verified again based on the expression levels of P53, P21 and CyclinD1 (Figure 4E).

### SENP1 regulates UBE2T through deSUMOylation

**Figure 5:**
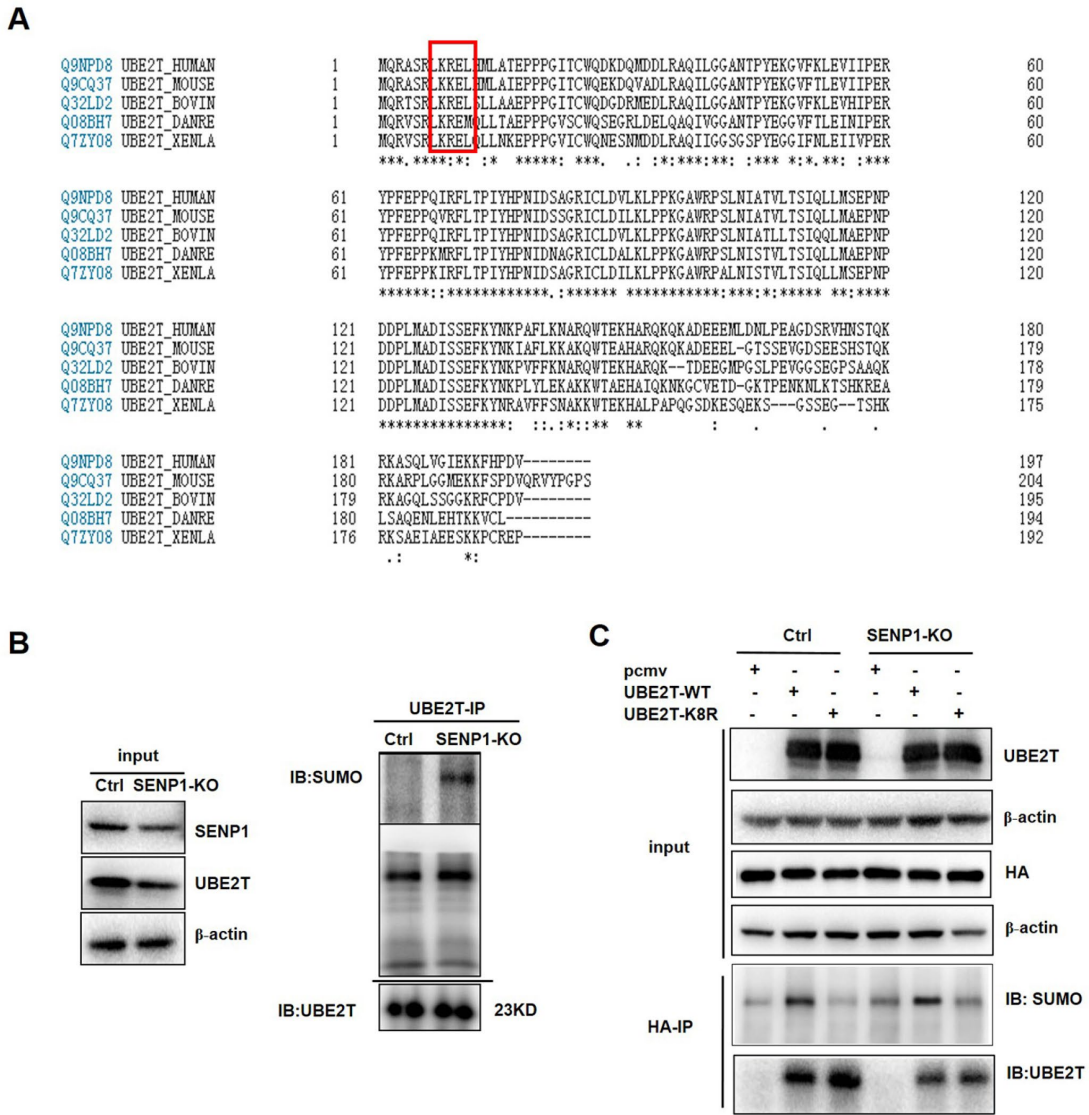
**SENP1 deSUMOylates UBE2T.** (**A**) Comparative analysis of UBE2T in different species. (**B**) The effect of SENP1 knockout on SUMO-UBE2T complex was determined using co-Immunoprecipitation. (**C**) The effect of UBE2T-overexpression and UBE2T-K8R mutation co-expressed with SENP1 knockout on SUMO-UBE2T complex was determined using co-Immunoprecipitation.

A commonly known SUMO acceptor site consists of the sequence ΨKXE (Ψ, a large hydrophobic amino acid; K, the site of SUMO conjugation; X, any amino acid) [16]. We found that UBE2T contains the SUMOylation site (KRE) *via* sequence alignment and bioinformatics analysis by the software SUMOplot and SUMOsp, thus we speculated that UBE2T could be SUMOylated [26] (Figure 5A). Therefore, we investigated whether the induction of UBE2T by SENP1 results from deSUMOylation. Our results showed that SENP1 knockout could enhance the SUMOylation of UBE2T *via* increasing SUMO-UBE2T complex (Figure 5B). Based on the fact that K8 site is the major acceptor sites for SUMOylation which is also proved to be universal *via* comparative analysis of UBE2T in different species (Figure 5A), we generated the mutated *UBE2T* gene (K8R) by which K was mutated to R to further determine SENP1-mediated deSUMOylation of UBE2T. PCMV-HA, PCMV-UBE2T-WT (wild-type), and a SUMO-defective mutant, PCMV-UBE2T-K8R were co-expressed with the negative control or SENP1 knockout group. As expected, SUMOylated UBE2T could be detected for UBE2T-WT, but not for the UBE2T-K8R, verifying the validity of the SUMOylation site (KRE) again. More importantly, the increased SUMOylation of UBE2T in SENP1 knockout group compared with the negative control proved the ability of SENP1 to deSUMOylate UBE2T (Figure 5C).

### Effect of UBE2T overexpression and K8R mutation on cell function and its downstream signaling pathway

**Figure 6:**
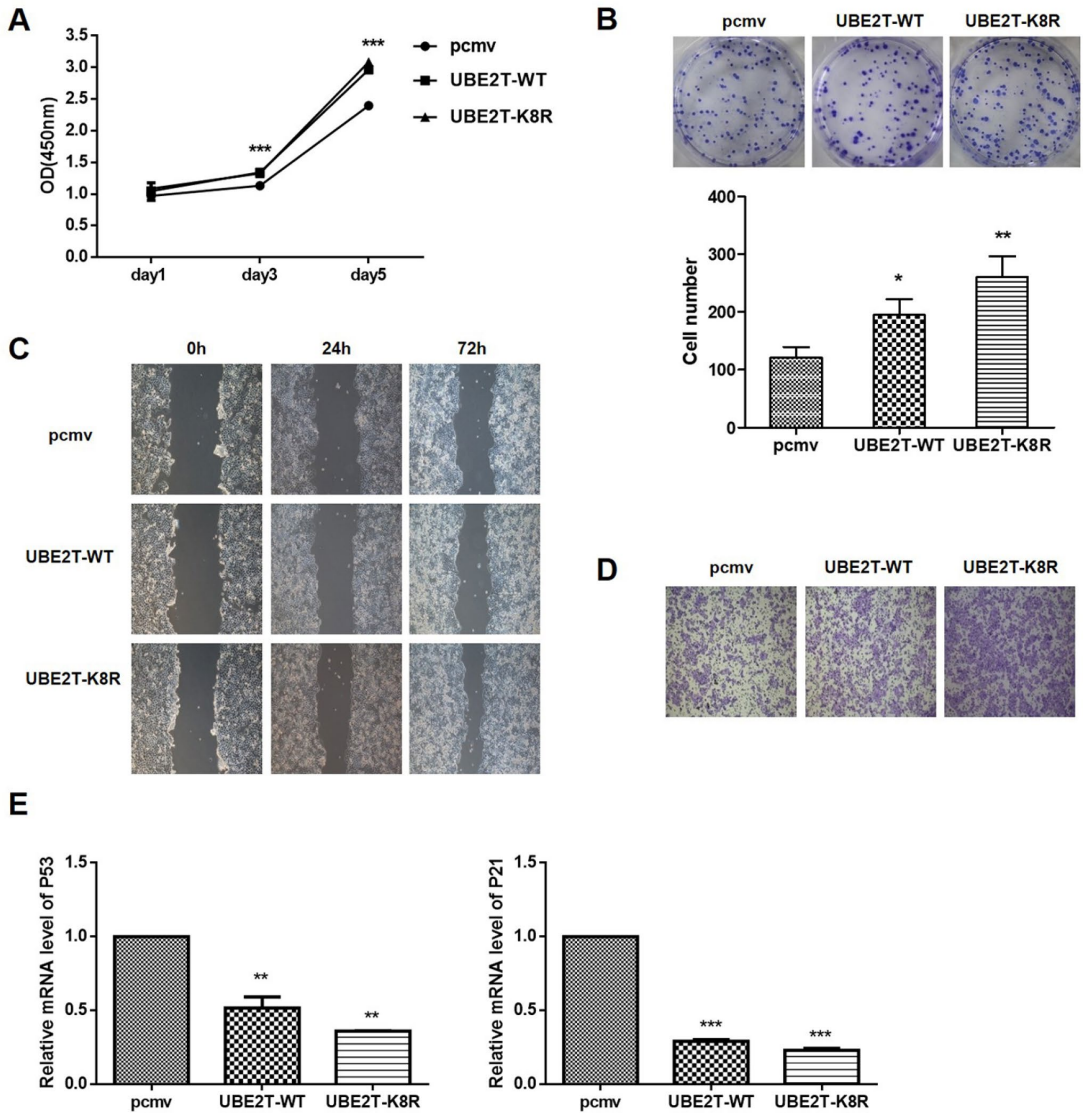
**Effect of UBE2T overexpression or K8R mutation on cell function and its downstream signaling pathway.** (**A**) The effects of UBE2T overexpression and K8R mutation on HepG2 cell proliferation were determined using CCK-8 assay. (**B**) The effects of UBE2T overexpression and K8R mutation on HepG2 cell colony formation were examined using colony formation assay. (**C**) The effects of UBE2T overexpression and K8R mutation on HepG2 cell migration were determined using wound healing assay. (**D**) The effects of UBE2T overexpression and K8R mutation on HepG2 cell invasion were examined using Transwell assay. (**E**) The effects of UBE2T overexpression and K8R mutation on p53 and p21 mRNA levels. The representative images were selected from at least three independent experiments. *P<0.05, **P<0.01, ***P<0.001.

To determine the effects of UBE2T overexpression and K8R mutation on cellular functions, HepG2 cells were transfected with PCMV-HA, PCMV-UBE2T-WT, and PCMV-UBE2T-K8R. As shown in Figure 6A and 6B, the overexpression and the K8R mutation of UBE2T have almost the same promotion effects on cell proliferation and colony formation. Wound healing assay and Transwell assay were performed to detect cell migration and invasion, which demonstrated that UBE2T overexpression and K8R mutation could both promote migration and invasion of HepG2 cells, in spite of slight differences (Figure 6C and 6D). Finally, mRNA levels of cell cycle related proteins P53 and P21, which were detected by RT-PCR, also showed obvious decrease in UBE2T-WT and UBE2T-K8R group compared with that in PCMV group (Figure 6E). Combining all the above mentioned experimental results, we found that overexpression and prevention of SUMOylation of UBE2T through K8R mutation have similar effect on HepG2 cells with SENP1.

### Effects of SENP1 Knockout on tumor growth *in vivo*

**Figure 7:**
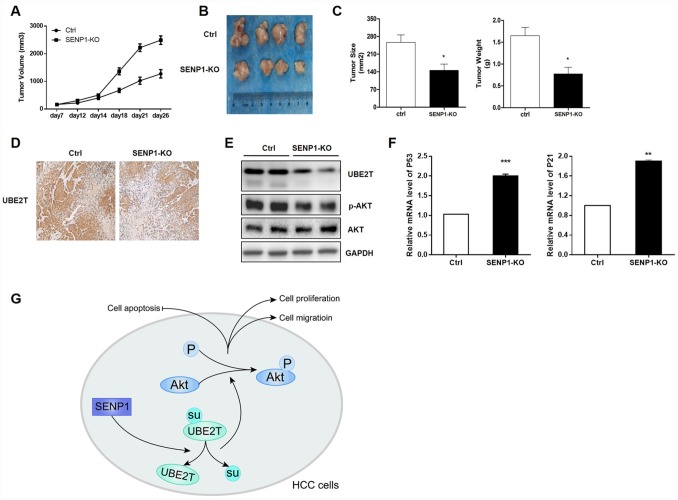
**SENP1 knockout attenuates the tumorigenic potential *in vivo*.** (**A**) Tumor growth of mice injected with HepG2-control or HepG2-SENP1 KO. (**B**) Representative tumors of negative control groups and SENP1 KO groups. (**C**) The average final tumor size and weight in negative control groups and SENP1-KO groups. (**D**) UBE2T expression in xenograft tumor tissues by immunohistochemical staining. (**E**) UBE2T, p-Akt and Akt protein levels in xenograft tumor tissues by Western blot. (**F**) P53 and P21 mRNA levels in xenograft tumor tissues by RT-PCR. (**G**) The schematic diagram briefly demonstrating the molecular mechanism of SENP1 mediated promotion of HCC. The representative images were selected from at least three independent experiments. *P<0.05, **P<0.01, ***P<0.001.

In order to verify the effects of SENP1 knockout on tumor growth *in vivo*, tumor-bearing mice model was constructed by injection of HepG2-SENP1-KO cells The trend of tumor growth showed significant slowdown in the SENP1-KO group than the negative control (Figure 7A). Accordingly, the final tumor size and weight in SENP1-KO group was significantly reduced compared with the negative control (Figure 7B and 7C). Therefore, this xenograft study verified the important role of SENP1 in driving tumor growth *in vivo*. Furthermore, the results of IHC and Western blot analysis confirmed the suppressed expression of UBE2T in SENP1-KO mice (Figure 7D and 7E). Meanwhile, is was also showed that SENP1 knockout blocked the activation of Akt (Figure 7E). In addition, the mRNA levels of cell cycle related proteins, P53 and P21 were increased in SENP1-KO group compared with control group (Figure 7F). Notably, all the results *in vivo* were in accordance with the *in vitro* results obtained before. Altogether, the molecular mechanism of SENP1 mediated promotion of HCC was summarized in Figure 7G.

## DISCUSSION

SUMOylation has emerged as an important regulatory mechanism for signal transduction and is involved in a variety of cellular processes such as transcription, nuclear transport, signal transduction, stress response and genome integrity [8, 27–31]. Currently, studies have revealed that cancer progression can be promoted by deregulation of either SUMO conjugation ordeconjugation [8, 27–31]. Considering that the majority of reports focused on the role of SUMOylation by the action of the conjugation enzyme Ubc9 or E3 ligases [32], the effect of the deSUMOylation still needs to be explored.

Among the seven SENP members, SENP1 was the first identified SUMO-specific protease. Overexpression of SENP1 has been observed in several cancer types, including colon cancer, proteases cancer, and breast cancer. Herein, we revealed enhanced SENP1 expression in most hepatocellular carcinoma tissues as compared with their adjacent normal tissues. Moreover, clinical data collected from 50 HCC patients demonstrated that the expression of SENP1 is associated with the severity of HCC, HCC aggressiveness and recurrence. Also, Kaplan-Meier analysis showed that HCC patients with relatively high SENP1 expression suffered from lower overall survival rate. Accordingly, clinical data indicated that patients with higher expression level of SENP1 generally suffered from larger tumor size, poorer histological characteristics, later TNM stage and more tumor number, which is also in accordance with the results of overall survival rate. Notably, the collected data from TCGA database also proved the upregulation of SENP1 in tumor tissues and the association of high SENP1 expression with lower overall survival rate. It was reported that silencing of SENP1 could reduce HCC cell proliferation and migration [33]. A similar role of SENP1 on tumor growth is also supported by our observation that knockout of SENP1 is linked to decreased HCC cell growth *in vitro* and *in vivo*, colony formation, migration and invasion. Furthermore, knockout of SENP1 could induce HCC cell cycle arrest. Regulation of cell cycle is linked to activation of CDKs (e.g. CDK1, CDK2, CDK4/6), which could be paired with Cyclins (e.g. Cyclins A, B, E, D1-3). Activation of CDKs was also antagonized by its inhibitors, such as P16, P19, P21 and P27 [11]. Here we showed that knockout of SENP1 resulted in downregulation of P53 and P21, and decreased expression of CyclinD1. Taken together, these results strongly proved that SENP1 promotes tumor development and progression of HCC. However, its targets and molecular mechanism is not clear completely.

In this study, we identify UBE2T as a novel target of SENP1 in HCC and discover the potential role of SENP1 in promoting UBE2T expression and deSUMOylation. UBE2T gene is located in 1q32.1, which was reported to be upregulated in HCC and promote HCC progression [19]. Consistently, our analysis revealed the high expression of UBE2T in most hepatocellular carcinoma tissues and HCC cell lines. More importantly, we illustrated the first report of the positive correlation between expression levels of SENP1 and UBE2T. Similar with the case of SENP1, it has been reported recently that the higher expression of UBE2T was accompanied with lower survival rate [34]. Again, our results were consistent with the data obtained from TCGA databased and KM plotter liver cancer dataset. Otherwise, K8 site has been recognized as the major acceptor sites for SUMOylation. Therefore, SUMOylation-resistant mutation (K8R) was constructed and illustrated to be more efficient than the wild-type protein in promoting cell proliferation, colony formation, migration and invasion.

To the best of our knowledge, this is the first report demonstrating that UBE2T could be deSUMOylated by SENP1. Although it is intriguing to suggest that SENP1 may act a significant role in tumor progression, few of its downstream targets have been identified so far. Our studies demonstrated that either UBE2T overexpression or UBE2T K8R mutation lead to attenuation of P53 and P21 levels. The fact that the prevention of SUMOylation of UBE2T (K8R mutation) has similar effect on properties of HepG2 cells with SENP1 suggests the deSUMOylation as the mechanism of SENP1 to induce UBE2T then the tumor progression.

Together with these findings, UBE2T and SENP1 have been shown to have many related functions in tumorigenesis of HCC, which has not been investigated together previously. Herein, our findings showed that both SENP1 and UBE2T were upregulated in either hepatoma cell lines or tumor tissues and their expression levels were positively related. Moreover, SENP1 knockout suppresses tumor development and progression while UBE2T overexpression or UBE2T K8R mutation in HCC cells displayed opposite impact. Therefore, the important discovery that UBE2T is deSUMOylated and activated by SENP1 suggests an arresting novel mechanism to promote UBE2T SUMOylation in human cancer.

## MATERIALS AND METHODS

### Patients and tissue specimens

50 pairs of HCC tissues and adjacent non-tumor tissues were obtained from the patients with HCC who underwent surgical resection. Samples were immediately snap-frozen in liquid nitrogen and stored at -80 °C. Informed consent was received from each patient, and this study was approved by the ethics committee of the department of Surgery and the hospital.

### Immunohistochemical staining

Immunohistochemistry was carried out by using SENP1, UBE2T and antibodies. In brief, formalin-fixed, paraffin-embedded tissues were cut into 4 μm thickness and subjected to deparaffinization and rehydration. Following antigen retrieval, tissue sections were incubated with primary antibody overnight at 4°C. After washed with PBS, sections were incubated with biotinylated secondary antibody for 1 h at room temperature. Diaminobenzidine (DAB kit) was employed to be as chromogen and slides were counterstained with hematoxylin.

### Cell lines and cell culture

Human liver cancer cell lines (Bel-7402, QGY-7701, Chang liver, SNU-423, SMMC-7721, LM3, MHCC97-L, MHCC97-H, HepG2) and the normal liver cell line LO2 were purchased from ATCC and cultured in DMEM supplemented with 10% FBS and 1% penicillin/streptomycin at 37 °C in a humidified incubator of 5% CO_2_.

### RNA isolation and real-time PCR

Total RNA from cells or tissue samples was isolated using Trizol reagent (Invitrogen). Quantitative real-time PCR analysis was performed with One Step SYBR PrimeScriptTM PLUS RT-PCR kit (Takara) using the ABI 7500 Real-Time PCR System (Applied Biosystems). GAPDH were used as an internal control. The relative expression was determined using the 2^−ΔΔCT^ method.

### Western blot and immunoprecipitation

For western blot, total protein from cells or tissue samples was extracted using RIPA lysis buffer (Beyotime, Shanghai, China), and protein concentration was measured by BCA assay (Beyotime, Shanghai, China). Equal amounts of protein were subjected to 10% SDS-PAGE, and transferred to PVDF membranes (Millipore, Billerica, MA, USA). For coIP, the protein extracts were incubated with antibody and Protein G-Sepharose (GE Healthcare). After blocking in 5% fat-free milk, the membranes were incubated with primary antibodies against different proteins overnight at 4 °C. Following incubation with HRP-conjugated secondary antibody for 2 h at room temperature, bands were visualized using the Enhanced Chemiluminescence system (Pierce). GAPDH or β-actin was used as internal reference. The intensity of protein bands was determined using the Image J software.

### Construction of Cas9-SENP1 cell line

The sgRNA (small guide RNA) construct, targeting SENP1, and the Cas9 expression construct, pRGEN-Cas9-CMV, were purchased from ToolGen (Seoul, Korea). The SENP1 exon located at the six coding exon was selected for guide RNA design. For the establishment of SENP1 knockout cell lines, HepG2 cells were transfected with sgRNA. The confirmation of the genome editing was performed using a GeneArt Genomic Cleavage Detection kit (Life technologies). SENP1 protein levels in sgRNA-transfected or control cells were demonstrated by Western blotting.

### Cell proliferation and colony-formation assay

For cell count assay, HepG2 cells were seeded at a density of 1×10^4^ cell/well in 96-well plates and cultured in DMEM supplemented with 10% FBS and 1% penicillin/streptomycin at 37°C in a humidified incubator of 5% CO_2_. Cell numbers were counted at the time point indicated in the relevant s legend. For CCK-8 assay, HepG2 cell were seeded at a density of 2,000 cell/well in 96-well plates and cultured in DMEM supplemented with 10% FBS and 1% penicillin/streptomycin at 37°C in a humidified incubator of 5% CO_2_. The cell proliferation was measured by CCK-8 assay according to manufacturer’s direction at the time point indicated in the relevant figure legend.

To measure colony-formation ability, cells were seeded at a density of 200 cell/well in six-well plates and cultured for 12 days. Colonies were then fixed with methanol and stained with crystal violet, followed by count of colonies.

### Cell migration assay

Cell migration ability was evaluated by using wound healing assay. Briefly, cells were seeded in six-well plates at a density of 5 × 10^4^ cell/well until confluent. After linear scratch wounds were created by 200 μL pipette tip, medium was replaced with serum-free medium and the cells were cultured for indicated times. Images were taken under a × 10 objective lens.

### Cell invasion assay

Cell invasive ability was examined by using Transwell chambers with 8 mm pore size. In brief, 5 × 10^4^ cells were placed in the upper chamber and cultured in serum-free DMEM. Medium with 10% FBS was added into the lower chamber. After incubation for indicated times, the invasive cells on the lower chamber were fixed with 4% paraformaldehyde and then stained with 0.5% crystal violet. The invasive cells were imaged by microscope at a magnification of 200 × in five random fields. Each experiment was repeated in triplicate.

### Cell cycle analysis

To determine cell cycle distribution, 1 × 10^6^ cells were fixed with 70% cold ethanol overnight at -20°C. After hydrolyzation with 250 μg/ml of RnaseA at 37°C for 30 min, cells were stained with 10 mg/mL propidium iodide at room temperature for 20 min. The samples were analyzed by FACScan flow cytometer (BD Biosciences) and the cell cycle distribution were calculated with CellQuest software (BD Biosciences). Each experiment was repeated in triplicate.

### Lentiviral infection

Human Lenti-UBE2T-Flag, Lenti-UBE2T-K8R-Flag were designed and purchased from Shanghai Bioscienceres, Co., Ltd (Shanghai China). The transfection was performed according to standard procedures.

### Analysis of tumorigenesis *in vivo*

BALB/C nude mice were purchased from Shanghai Bikai Lab Animal Co., Ltd. For the tumorigenesis assays *in vivo*, 5 × 10^6^ HepG2-control cells or HepG2-SENP1-KO cells were subcutaneously injected into the flanks of male BALB/c-nude mice (n = 5 per group, 4-week old). Tumor growth was measured with calipers from day 7 to day 26 after injection and the tumor volume was calculated using the following formula: tumor volume = [length×width^2^]/2. Mice were sacrificed at 26 days after injection, and tumors were dissected and weighted. All experimental protocols using mice were approved by the Ethics Committee of Experimental Animals of Fudan University and all experiments also conformed to the guidelines of the Chinese Association of Laboratory Animals.

### Statistical analysis

All the data are expressed as the mean ± SD from at least three independent experiments. Statistical analysis was performed by using the GraphPad Prism 6.0 software. The differences between groups were analyzed using Student’s test for two groups or by one-way of variance (ANOVA) followed by Dunnet t-test for multiple groups. *P* < 0.05 was considered statistically significant. Expression of SENP1 and UBE2T in normal and tumor tissues were analyzed with Pearson correlation by GraphPad Prism 6.0.
